# Gravesend Hospital and the War

**Published:** 1914-12-19

**Authors:** 


### GRAVESEND HOSPITAL AND THE WAR.

### Mr. W. Pearson and Miss Davidson on its Work.

Many accounts have appeared in The Hospital, thanks to the co-operation of our special correspondents, of the arrival of the wounded and the rush of work entailed on the day of emergency, but up to the present no summary of the situation from the outbreak of war has been published. The experiences of one of the smaller institutions are available this week through the courtesy of Mr. Walter Pearson, Secretary of the Gravesend Hospital.

"Our experiences at Gravesend," Mr. Pearson began in a preliminary talk, "are chiefly interesting because they give some idea of the way in which one of the smaller institutions, being determined to do what it could at the very start, rose to the occasion, and by preparation in advance was able to cope with an access of work far beyond its normal capacity. We endeavoured to do our duty both to civilians and soldiers by raising the number of beds in the wards, and by placing up to fourteen beds, in case of emergency, in the out-patient department."

"How did you meet the increased demand for funds and stores?"

"Taking the stores first, the people in the district came forward and provided all the necessary linen and either gave or loaned the necessary extra beds. The result was that none of the general funds of the hospital had to be expended on these things. Do not, however, suppose that it was so much extra money which we received. Sheets and bedding were given, and, in addition, pieces of linen, which, with our own store, Matron was able to hand out to volunteers, among whom stitching and sewing parties became the order of the day. Thanks chiefly to Mrs. Snelling, the Chairwoman, we have a very flourishing Ladies' Association, which provides not merely linen, but a body of willing workers actively interested in the hospital in all sorts of ways. As to the cost of maintaining the soldiers, we are hoping to receive a sum almost equivalent to the average cost per patient per day for each soldier through the Paymaster-General, for this appears to prove acceptable to the authorities, and a monthly account will be rendered."

The questions to Miss Davidson, the matron, resolved themselves into this: What is the value of volunteer probationers, and how do they fit in in practice with the ordinary administration of her department?

"They have justified themselves," Miss Davidson replied without hesitation; "and I attribute this mainly to the fact that our volunteer probationers were all drawn from ladies whose interest in the hospital was shown long before the war! Of these tried friends I organised a corps of twenty volunteers, pro-probationers, who came into the hospital at once to complete their knowledge of the geography of the place, and they soon became, when the rush of wounded arrived, extremely useful. They were warned that nursing as such and first-aid was not what was required of them, but practically without exception they settled down cheerfully to such humble but essential work as cutting bread and butter, serving ward meals, some cleaning and washing, running messages, and doing what they were asked to do generally. At all events, they felt themselves soon sufficiently a part of the hospital to be heard to speak of 'my' ward, and to tell their friends that they had now been 'put on night duty.' The experiment has been quite successful, partly, as I say, because it was not 'war enthusiasts' who were chosen, and partly because it was carefully explained to them at the start what sort of things—for instance, nursing—they would *not* be expected to do."

Mr. Pearson then led the way round the institution.

"I think you will be interested," he said on approaching the out-patient department, "in the sanitary unit, devised for the occasion. There were, of course, two closets for the use of outpatients, and two roomy cupboards. The latter have been used as store-rooms for ward crockery, bed-pans, and so on, and the closets are also enabled to perform some of the functions of a sink-room, thanks to the fitting of a device which I saw advertised in The Hospital a little while ago. This is the bracket bed-pan washing apparatus, manufactured by Morrison, Ingram and Co., of Manchester, for washing bed-pans over an ordinary water-closet. Being on a swivel it can be set back when not in use, and when used only projects eighteen inches from the wall. I am bound to say that it has certainly helped us out of a difficulty and extended the possibilities of our water-closets in this extemporised ward very usefully. The whole department can be fitted up as a ward with the help of our two porters in twenty minutes. The arrival of the wounded has been described more than once in The Hospital, so I need not go into that now."

